# Transparent and ‘opaque’ conducting electrodes for ultra-thin highly-efficient near-field thermophotovoltaic cells

**DOI:** 10.1038/s41598-017-13540-8

**Published:** 2017-10-25

**Authors:** Aristeidis Karalis, J. D. Joannopoulos

**Affiliations:** 1 Research Laboratory of Electronics, Massachusetts Institute of Technology, Cambridge, MA 02139 USA; 2 Department of Physics, Massachusetts Institute of Technology, Cambridge, MA 02139 USA

## Abstract

Transparent conducting electrodes play a fundamental role in far-field PhotoVoltaic systems, but have never been thoroughly investigated for near-field applications. Here we show, in the context of near-field planar ultra-thin ThermoPhotoVoltaic cells using surface-plasmon-polariton thermal emitters, that the resonant nature of the nanophotonic system significantly alters the design criteria for the necessary conducting front electrode. The traditional ratio of optical-to-DC conductivities is alone not an adequate figure of merit, instead the desired impedance matching between the emitter and absorber modes along with their coupling to the free-carrier resonance of the front electrode are key for optimal device design and performance. Moreover, we demonstrate that conducting electrodes ‘opaque’ to incoming far-field radiation can, in fact, be used in the near field with decent performance by taking advantage of evanescent photon tunneling from the emitter to the absorber. Finally, we identify and compare appropriate tunable-by-doping materials for front electrodes in near-field ThermoPhotoVoltaics, specifically molybdenum-doped indium oxide, dysprosium-doped cadmium oxide, graphene and diffused semiconductors, but also for ‘opaque’ electrodes, tin-doped indium oxide and silver nano-films. Predicted estimated performances include output power density ~10 *W*/*cm* ^2^ with >45% efficiency at 2100 °*K* emitter temperature and 60 Ω electrode square resistance, thus increasing the promise for high-performance practical devices.

## Introduction

Near-field ThermoPhotoVoltaics (TPV) is a recent exciting technology^1–4^, promising to deliver high-power-density generators, which can be powered by numerous sources (hydrocarbon fuels, nuclear reactors, solar irradiation, waste industrial heat etc.) and can also be light, portable and involve no internal mechanical motion. Their principle of operation relies on channeling heat to an emitter and thereby thermally exciting evanescent photons, which are decaying through a vacuum gap into a *nm*-spaced PhotoVoltaic (PV) cell, where they are absorbed and converted to electricity. Various flavors of such systems have been proposed, including Surface-Plasmon-Polariton (SPP) emitters with bulk PV-cell absorbers without^5–8^ and with^4,9^ a metal back-surface reflector across few-*nm*-sized gaps and semiconductor emitters with thin-film PV cells over wider spacings^10^. In a recent work^11^, we highlighted the large degree to which absorption by the free carriers of the required PV-cell front electrode can influence the system efficiency and we proposed a system topology and design, which minimizes these losses, by resonantly cross-coupling and impedance-matching^12^ an emitter SPP state with an absorber single photonic state, thus accomplishing narrowband near-field emission just above the semiconductor bandgap and across an ample vacuum spacing. An important component missing from all of these promising near-field TPV studies is a thorough investigation of front conducting-electrode design requirements for optimal performance.

Transparent conducting electrodes (TCE) play a fundamental role in regular far-field PhotoVoltaics, especially solar cells, and in many optical LEDs, such as flat panel displays. Therefore, extensive analyses of materials and design methods are available for decades now^13^. Since optical absorption and DC conduction both depend on the free carriers of the electrode, the main TCE performance trade-off lies in achieving low-resistance electrical conduction without sacrificing transmission in the visible range of the radiation crossing the electrode. Therefore, the far-field TCE performance can be characterized by a single figure of merit, the ratio of optical-to-DC conductivities of the electrode material, and thus the design boils down basically to material selection, with Indium Tin Oxide (ITO) the material of choice for most modern applications^13^.

However, the design principles for conducting electrodes employable to near-field (T)PV systems have never been carefully studied, to our knowledge, and this is the task of this article. For the evanescent fields involved in near-field cells, reflection is luckily not a problem, but the trade-off between DC conduction and optical absorption remains. Certainly, material selection will again play an important role and we do identify and compare low-loss materials that are conducting but transparent in the infrared and thus appropriate for TPV-cell electrodes. However, we also introduce concepts that are new for near-field systems and do not carry over from traditional far-field conducting-electrode design. Specifically, we demonstrate that a single figure of merit is not always enough to evaluate a near-field electrode performance and we show that even electrodes that are ‘opaque’ to far-field radiation can actually efficiently ‘transmit’ evanescent waves. Our numerical analysis involves realistic material modeling, exact semi-analytical electromagnetic calculations, albeit simplified electronic modeling, followed by topology optimizations, so that we can draw comparative conclusions among optimal structures. Such an analysis is appropriate given, as we shall see, that photonic losses matter significantly and are key in determining the performance of our coupled near-field TPV system designs.

## Results

### Proposed structure and front-electrode square resistance

**Figure 1:**
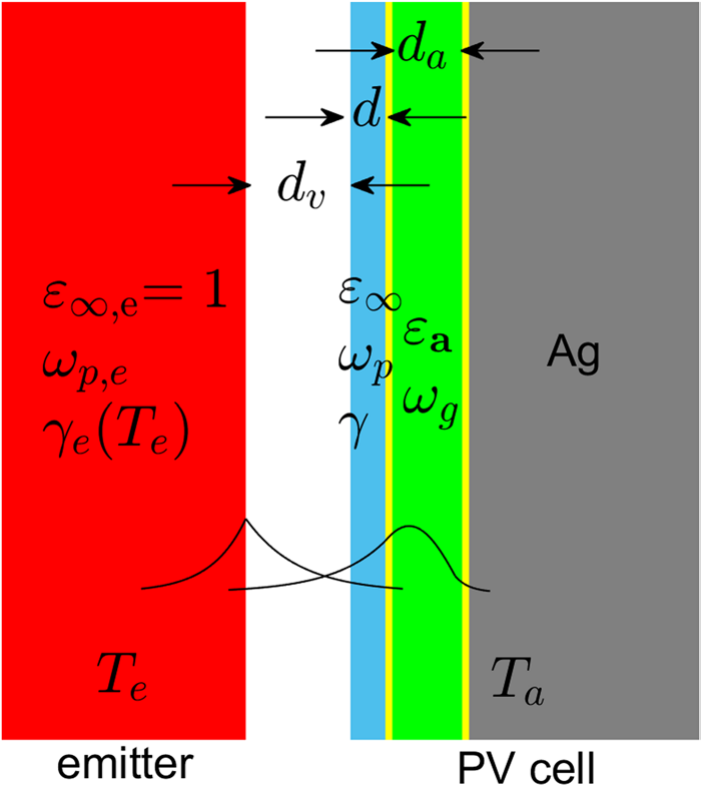
Near-field TPV structure consisting of a plasmonic emitter (red), supporting a SPP mode, and a PV cell, supporting a single confined photonic mode at the bandgap frequency, and made of a thin-film semiconductor absorber (green), a silver back electrode (gray) and the front electrode under examination (blue). The amplitudes of the two modes and their coupling inside the vacuum gap are also shown qualitatively.

The typical proposed planar TPV system is shown in Fig. 1. It consists of a plasmonic emitter *e*, at a high temperature *T*_*e*_, supporting a SPP state on its interface with the vacuum gap, and an ultra-thin PV-cell absorber *a*, at *T*_*a*_ < *T*_*e*_. The PV cell is comprised by an ultra-thin film of semiconductor material, supporting a single guided photonic state at the frequency of its electronic bandgap *E*_*g*_, backed by silver, functioning as the back electrode and removing photonic modes below the bandgap^11^, and fronted by an ultra-thin layer of a conducting material, which functions as the front electrode but allows the hot photons to pass through. In all designs in the present article, the semiconductor material is chosen so that its bandgap scales with the emitter temperature as *E*_*g*_ = 4 *k*_*B*_ *T*_*e*_, to maximize emitted power *per surface area* ${P}_{e}\sim {T}_{e}^{4}$ and to enhance efficiency^11^. The photonic system is designed such that the emitter SPP mode and the absorber photonic mode are cross-coupled and impedance-matched just above *E*_*g*_ to optimize efficiency^11,12^.

Since the semiconductor film is only single-mode thin, we will assume in this article that its pn-junction depletion (space-charge) region extends throughout it and an electric field (potential *V*) is built in across it (see more in Discussion and Methods later). Therefore, electrons and holes photo-generated via inter-band photon absorption inside the semiconductor will be swept out by its built-in potential *V* into the back and front electrodes. In a real device, the front-electrode layer will typically be assisted by a grid of metallic nano-wires, either printed on top of the layer or buried inside it. Entering this ultra-thin front-electrode layer, the carriers will therefore “turn” to run parallel to it and try to reach the more-conducting metallic nano-wires. This lateral current will cause resistive losses inside the front electrode. Its desired square resistance *R*_*sq*_ = 1/*σ*_*DC*_ *d* will determine how high the DC conductivity *σ*_*DC*_ and how large the thickness *d* of the thin-film electrode have to be. The conductivity can be calculated using the Drude model for free-carriers: we equate the relative permittivity of Eq. (7) to *ε*_∞_ + i*σ*(*ω*)/*ωε*_*o*_ to find

1 $$\sigma (\omega )=\frac{{\varepsilon }_{o}{\varepsilon }_{\infty }{\omega }_{p}^{2}}{\gamma -{\rm{i}}\omega }\Rightarrow {\sigma }_{DC}=\sigma (\omega =0)=\frac{{\varepsilon }_{o}{\varepsilon }_{\infty }{\omega }_{p}^{2}}{\gamma }=\frac{{\varepsilon }_{\infty }{\omega }_{p}^{2}}{{Z}_{o}c\gamma }.$$

where ${Z}_{o}=\sqrt{{\mu }_{o}/{\varepsilon }_{o}}$ the impedance of free space. For each candidate electrode material the mobility *μ* of free carriers depends on their density *N*; since *γ* = *q*/*μ*(*N*)m* and ${\omega }_{p}=q\sqrt{N/{\varepsilon }_{o}{\varepsilon }_{\infty }{m}^{\ast }}$, each electrode material can be described either by *μ*(*N*) or, equivalently, by a function *γ*(*ω*_*p*_), where *N* is an underlying tunable-via-doping parameter. A given desired electrode square-resistance imposes then the condition

2 $${R}_{sq}=\frac{1}{{\sigma }_{DC}d}=\frac{1}{q\mu (N)Nd}={Z}_{o}\frac{c\gamma ({\omega }_{p})}{{\varepsilon }_{\infty }{\omega }_{p}^{2}d}\iff d=\frac{{Z}_{o}}{{R}_{sq}}\frac{c\gamma ({\omega }_{p})}{{\varepsilon }_{\infty }{\omega }_{p}^{2}}$$

### Performance dependence on front-electrode doping

Typically, *γ*(*ω*_*p*_) scales slower than ~${\omega }_{p}^{2}$, so the higher the carrier density *N*, the more conductive the thin-film electrode material is, and therefore the thinner it needs to be. However, a material with high DC conductivity *σ*_*DC*_ will typically also have high optical conductivity Re{*σ*(*ω*)}, thus absorbing more impingent photons, instead of allowing them through. The ratio Re{*σ*_*Optical*_}/*σ*_*DC*_ is widely accepted as the fundamental figure of merit for solar-cell transparent conducting electrodes^13^. Using Eq. (1),

3 $${\rm{R}}{\rm{e}}\{\sigma (\omega )\}=\frac{{\varepsilon }_{o}{\varepsilon }_{{\rm{\infty }}}{\omega }_{p}^{2}\gamma }{{\gamma }^{2}+{\omega }^{2}}\Rightarrow \frac{{\rm{R}}{\rm{e}}\{\sigma (\omega )\}}{\sigma (0)}=\frac{{\gamma }^{2}}{{\gamma }^{2}+{\omega }^{2}}$$

Therefore, to minimize this ratio, one typically choses the electrode material and doping level that give the smallest possible loss rate *γ*(*ω*_*p*_), provided *ω*_*p*_ is smaller than the operating frequency range, so that the conducting electrode is, in fact, transparent (Re{*ε*(*ω* ≥ *ω*_*p*_)} > 0). One may then easily be tempted to conclude that the same design principles apply for near-field TPV systems. However, we will show here that the situation can actually be different.

Eq. (2) poses an additional restriction, on the necessary thickness of the thin-film front electrode. Furthermore, the free carriers on the front electrode induce, on its interface with the vacuum gap, a SPP mode, whose frequency is affected by the precise value of front-electrode free-carrier plasma frequency *ω*_*p*_^11^. For far-field PV systems, both these effects are present but they hardly affect performance, as they are largely irrelevant to the incoming far-field radiation: the electrode thickness only weakly influences the transmittance of propagating photons, which also do not couple to the front-electrode SPP mode below the light line. In near-field thin-film TPV though, the electrode thickness affects the key evanescent coupling between the emitter SPP and the semiconductor single mode in an exponential way. Moreover, the front-electrode *ω*_*p*_ dictates the frequency of its SPP, whose direct coupling to the emitter SPP photons and their subsequent free-carrier absorption was shown in ref.^11^ to be an important limiting factor of efficiency. Certainly, given a free-carrier plasma frequency *ω*_*p*_, indeed the electrode material choice with minimum *γ* (namely least possible carrier scattering rates and therefore highest mobility) will likely be the best, as it will also lead to the thinnest electrode and the smallest absorption in its free carriers. However, given a certain electrode material, it is not straightforward that one should choose the doping level (*ω*_*p*_) that minimizes *γ*, as the ratio Re{*σ*_*Optical*_}/*σ*_*DC*_ alone is not a sufficient figure of merit anymore.

**Figure 2:**
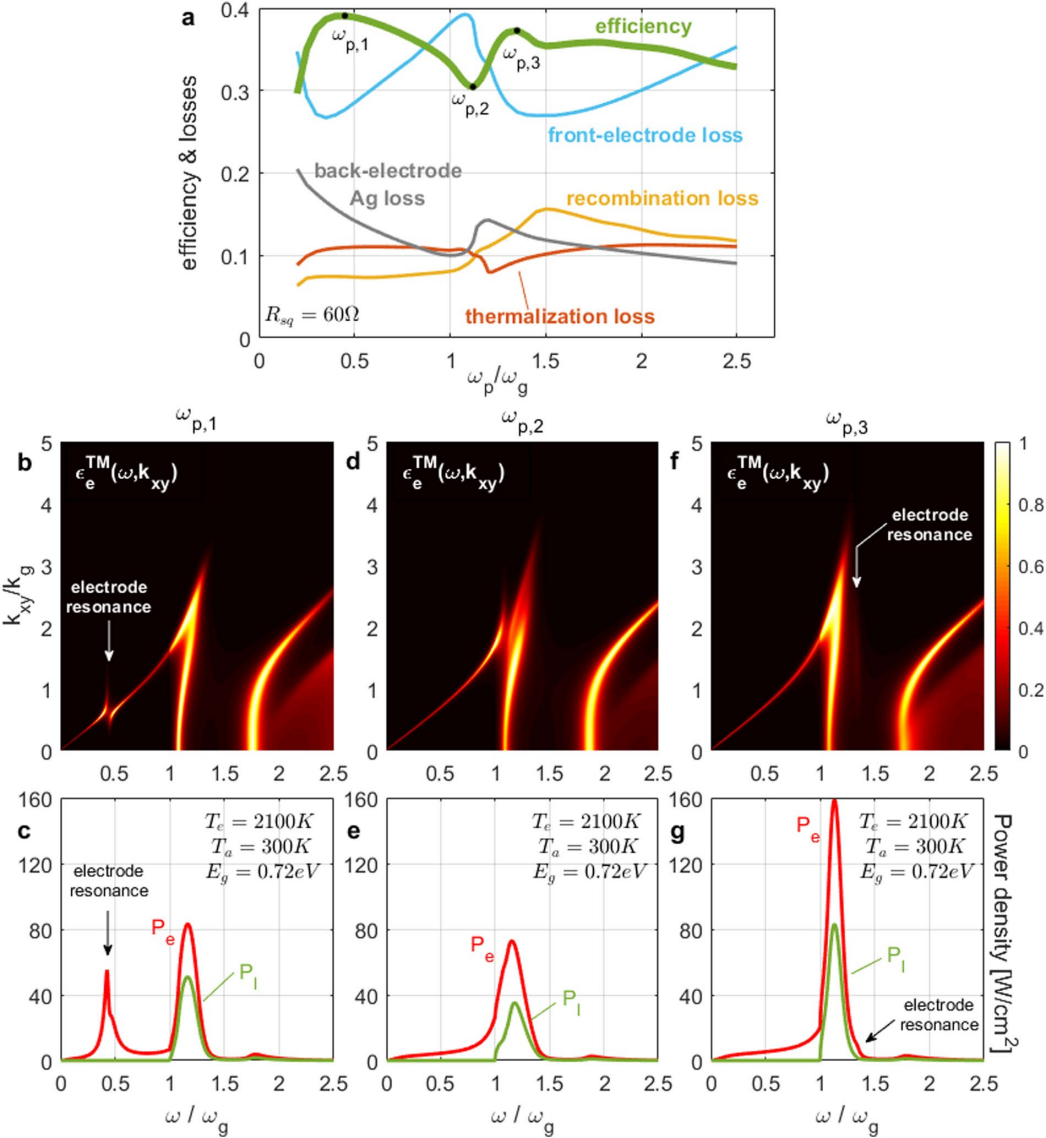
Optimization results vs doping level *ω*_*p*_ for the structure of Fig. 1 with model electrode *ħγ* = 0.0072 *eV* + 0.04 *ħω*_*p*_, at *T*_*e*_ = 2100 °*K*, *T*_*a*_ = 300 °*K*, *R*_*sq*_ = 60Ω and with *E*_*g*_ = 4 *k*_*B*_ *T*_*e*_ = 0.72 *eV*. (**a**) Efficiency *η* and losses [thermalization ~1 − *E*_*g*_/*ħω* per absorbed photon and recombination = (*E*_*g*_/*qV* − 1)*η*]. (**b**–**g**) Spectra at 3 doping levels *ω*_*p*_ indicated on (**a**) with black dots: (**b**,**c**) *ω*_*p*,1_, (**d**,**e**) *ω*_*p*,2_, (**f**,**g**) *ω*_*p*,3_. (**b**,**d**,**f**) TM emitter emissivity ${\epsilon }_{e}(\omega ,{k}_{xy})$. (**c**,**e**,**g**) TM emitter-power *P*_*e*_(*ω*) (red line) and load-power *P*_*l*_(*ω*) (green line) densities per surface area at the optimal-efficiency load voltage. Note that at *ω*_*p*,3_ the electrode is actually ‘opaque’ (i.e. Re{*ε*(*ω*_*g*_)} < 0) but thin enough for photons to tunnel through.

Real electrode materials, each with its own real mobility dependence on doping density, will be examined in a later section. However, as a first step to get a purely *qualitative* understanding of the effect of the front-electrode doping level on our ultra-thin near-field TPV system, we consider a model material with permittivity *ε* described by the Drude model of Eq. (7) with *ε*_∞_ = 4 and the simplified heuristic assumption that, as doping (*ω*_*p*_) varies, the loss rate scales linearly as *ħγ* = 0.0072 *eV* + 0.04 *ħω*_*p*_. This scaling is based on the reasoning that room-temperature mobility for some semiconductors roughly scales as $\mu \sim 1/\sqrt{N}$ at high doping due to ionized-impurity scattering and reaches a finite value at zero doping due to acoustic- and optical-phonon scattering, while the values are motivated by the properties of real materials (Fig. 4a), to which qualitative conclusions of the present analysis will also apply, as we shall see later. The results of TPV-efficiency optimizations (see Methods) for every value *ω*_*p*_ of this model material, at ${T}_{e}=2100\,^\circ K\Rightarrow {E}_{g}=4\,{k}_{B}{T}_{e}\approx 0.72\,eV$, *T*_*a*_ = 300 °*K* and for a fixed *R*_*sq*_ = 60Ω, are shown in Fig. 2a for the optimized efficiency and various losses, and in the “Supplementary Information” Fig. S1 for the optimal parameters. In Fig. 2b–g, we show the optimized TM emitter emissivity spectrum ${\epsilon }_{e}^{TM}(\omega ,{k}_{xy})$ (defined in ref.^11^) and emitter/load power densities for 3 doping levels *ω*_*p*1_ − *ω*_*p*3_.

The standard far-field arguments of wanting a conducting electrode, which is transparent (*ω*_*p*_ small enough) and with minimal Re{*σ*_*Optical*_}/*σ*_*DC*_ (so minimal *γ*), would show efficiency *monotonically decreasing* with *ω*_*p*_. However, Fig. 2a shows interesting dependence of efficiency on *ω*_*p*_, greatly departing from this monotonic behavior. Furthermore, it indicates that this efficiency variation is highly correlated (namely mainly due) to the losses at the front-electrode. Maximum efficiency is observed at an *ω*_*p*1_ < *ω*_*g*_, such that the electrode is indeed transparent at and above *ω*_*g*_ (Re{*ε*(*ω* ≥ *ω*_*g*_)} > 0). The corresponding TM emitter emissivity spectrum (Fig. 2b) indicates that hot photons are absorbed by the SPP mode formed on the electrode-vacuum interface at a frequency ~*ω*_*p*1_ below *ω*_*g*_, with an associated peak in emitted (but lost) power in Fig. 2c. For smaller values of *ω*_*p*_ < *ω*_*p*1_, the electrode thickness *d* must increase (according to Eq. (2) to get the fixed specified *R*_*sq*_) so much that, to maintain the necessary coupling and impedance-matching between the absorber single mode (confined in the semiconductor) and the emitter SPP, the vacuum-gap width *d*_*v*_ has to decrease significantly (Fig. S1b). This would also boost the undesired direct coupling of emitter photons to the free-carrier absorption losses of the front and back electrodes, so their associated losses are seen to worsen in Fig. 2a and efficiency decreases, even though *γ*(*ω*_*p*_) decreases in that direction. As *ω*_*p*_ increases above *ω*_*p*1_, the front-electrode SPP moves closer to the bandgap and its associated free-carrier absorption loss increases, reaching a system-efficiency minimum at ${\omega }_{p2}\mathop{ > }\limits_{ \tilde {}}{\omega }_{g}$, where a triple resonance is observed among the emitter SPP mode, the front-electrode SPP mode and the absorber photonic mode (Fig. 2d) and thus more emitter power is transmitted to the lossy carriers. This triple resonance phenomenon would not be observed for a far-field implementation (large *d*_*v*_). At *ω*_*p*3_ > *ω*_*g*_, another peak in efficiency is observed. The electrode is then, in fact, ‘opaque’ at *ω*_*g*_ (Re{*ε*(*ω*_*g*_)} < 0), but it is ultra-thin, so the hot evanescent photons tunnel through it. Such an ‘opaque’ conducting electrode is a novel concept that can only really exist in near-field (T)PV, as evanescent modes are of the essence for its functionality. The triple resonance is avoided, with the front-electrode SPP appearing at a frequency enough above *ω*_*g*_ (Fig. 2e), so the efficiency is high, but it is slightly lower than at *ω*_*p*1_, mainly because *γ* is higher. For the same reason and also because the vacuum gap has to shrink for adequate tunneling, the efficiency decreases for *ω*_*p*_ > *ω*_*p*3_ and the front-electrode losses deteriorate. However, the output power is much higher, which can be useful, if the small vacuum gap can be maintained.

A noteworthy remark is that, for all values of *ω*_*p*_, the optimized structures have electrode and depletion-region widths, such that the first (*k*_*xy*_ = 0) cutoff of the system photonic modes lies at a frequency slightly above the bandgap (Fig. 2b,d,f), as should be expected to avoid below-bandgap transmission^11^. Consequently, the transmitted power has a narrow spectrum slightly (~10%) higher than *E*_*g*_ (Fig. 2c,e,g), so the thermalization losses (~1 − *E*_*g*_/*ħω* per absorbed photon) are fairly constant (~10%) with *ω*_*p*_ (Fig. 2a).

In conclusion, for ultra-thin single-mode PV cells in the near field of a (thermal) emitter, the front electrode needs to have sufficient doping to ensure that it is itself ultra thin and emitter-absorber impedance matching is maintained with an ample vacuum gap. The doping level should be such that the electrode is preferably transparent at the bandgap frequency, but it can also function efficiently if opaque, via evanescent-field tunneling, while a (triple) resonance of the electrode carriers with the emitter and absorber modes should typically be avoided.

### Performance dependence on front-electrode square resistance and on temperature

The appropriate value of the front-electrode square resistance *R*_*sq*_ depends on the amount of resistive loss the designer is willing to tolerate. In the simple but common case where the metallic nano-wire grid assisting the front electrode consists of linear parallel nano-wires spaced by 2*w*, the efficiency drop due to the finite *R*_*sq*_ can be estimated by^14^ Δ*η*/*η* = *R*_*sq*_ *Iw* ^2^/3*V*, where *I* = *P*_*l*_/*V* is the PV-cell output current *per surface area*. For example, the design of Fig. 2b,c at *ω*_*p*1_ with a nano-wire spacing 2*w* = 100 *μm* would suffer losses Δ*η*/*η* ≈ 1%. For different grid spacings or system specifications, different values of the square resistance may be more appropriate.

**Figure 3:**
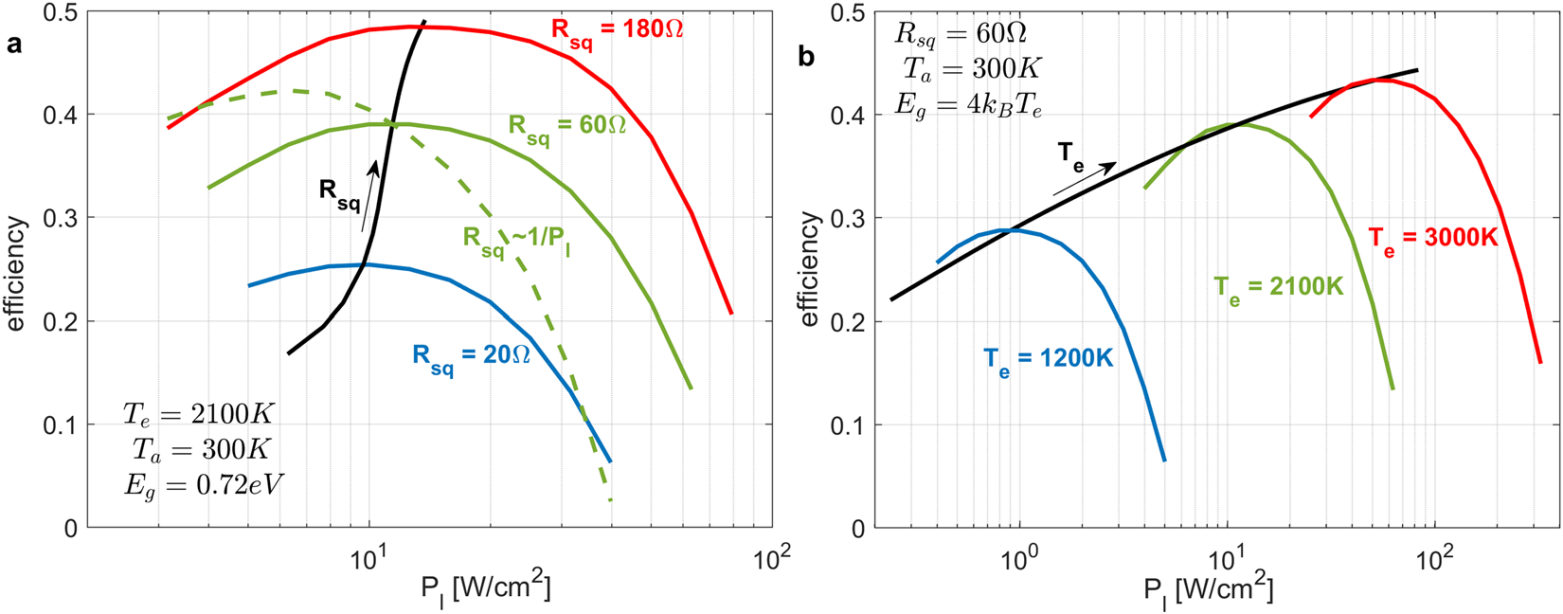
Optimized efficiency vs load power per surface area *P*_*l*_ for the structure of Fig. 1 with model electrode *ħγ* = 0.0072 *eV* + 0.04 *ħω*_*p*_, at *T*_*a*_ = 300 °*K* and with *E*_*g*_ = 4 *k*_*B*_ *T*_*e*_. (**a**) For 3 values of electrode square resistance *R*_*sq*_ at *T*_*e*_ = 2100 °*K*; black line shows the peak efficiency possible as *R*_*sq*_ varies; green dashed line assumes that *R*_*sq*_ scales inversely to *P*_*l*_, so that electrical losses Δ*η*/*η* are roughly constant. (**b**) For 3 values of emitter temperature *T*_*e*_ at *R*_*sq*_ = 60Ω; black line shows the peak efficiency possible as *T*_*e*_ varies.

The standard way to evaluate power converters is to plot efficiency versus power level. This is even more appropriate for a PV power conversion system, which has an inherent trade-off between efficiency and power output. In Fig. 3a, we show the maximum achievable conversion efficiency (not accounting for Δ*η*/*η*) as a function of the desired output power *per surface area P*_*l*_ (obtained using constrained optimization) at *T*_*e*_ = 2100 °*K* and for 3 different values of *R*_*sq*_, while the corresponding optimized parameters are shown in “Supplementary Information” Fig. S2. Higher power is accomplished mainly by decreasing the vacuum gap (Fig. S2b) and thus impedance matching the emitter and absorber modes at a higher in-plane wavevector *k*_*xy*_; then efficiency steadily decreases, as the sharp-decaying emitter fields cannot penetrate deep enough through the front electrode into the depletion region. (In contrast, in ref.^11^, the efficiency was seen to asymptote to some value at high power levels, due to the fact that the electrode and depletion regions of the PV cell were not physically separated.) A lower *R*_*sq*_ is achieved optimally by increasing both the thickness and doping of the front electrode (Fig. S2c,d) and, as expected, leads to lower efficiency due to increased free-carrier absorption losses inside it. For each curve, there is an output power value, for which the efficiency is maximized and those efficiency maxima are also plotted versus *R*_*sq*_ in Fig. 3a. Note, however, that, along each such constant-*R*_*sq*_ curve, the amount of additional resistive loss expected is varying, since Δ*η*/*η* ~ *R*_*sq*_ *I* ~ *R*_*sq*_ *P*_*l*_. To keep this loss roughly constant at all power levels, we also show the optimized efficiency, when *R*_*sq*_ is scaled as ~1/*P*_*l*_. The peak efficiency is not-surprisingly shifted to lower power levels. A crucial conclusion of this analysis is that the efficiency of a near-field TPV system depends greatly on the square resistance of the front electrode, therefore its modeling is absolutely necessary to make any realistic performance estimations.

It is instructive to also examine how the photonic design and performance depend on the emitter temperature *T*_*e*_. In Fig. 3b, optimized efficiency versus desired output power is plotted again at a constant *R*_*sq*_ = 60Ω but for 3 different emitter temperatures, and the corresponding parameters are shown in “Supplementary Information” Fig. S3. The main two effects are the scalings of the efficiency following the Carnot limit *η* ~ 1 − *T*_*a*_/*T*_*e*_ and of the output power as ${P}_{l}\sim {T}_{e}^{4}$ due to the choice *E*_*g*_ = 4 *k*_*B*_ *T*_*e*_. From Fig. S3, one can see that, if we normalize to this power scaling, the optimal layered-system thicknesses and the emitter and front-electrode materials plasma wavelengths scale roughly as ~*λ*_*g*_ = *hc*/*E*_*g*_ ~ 1/*T*_*e*_. Moreover, since $I\sim {T}_{e}^{3}$ and the efficiency-optimal voltage is typically close to the value^11^ *qV* ≈ *E*_*g*_(1 − *T*_*a*_/*T*_*e*_) ~ *T*_*e*_ for ${T}_{e}\gg {T}_{a}$, the efficiency drop Δ*η*/*η* can be relatively independent of emitter temperature with the same *R*_*sq*_, if also *w* ~ *λ*_*g*_ ~ 1/*T*_*e*_, namely if the geometrical scaling applies to all dimensions. Finally, Fig. 3b indicates that, if lower power is required than the max-efficiency point, for a small power range, it is preferable to reduce the power by increasing the vacuum gap (Fig. S3b), but, for much lower power, one should rather reduce the emitter temperature.

### Performance comparison of realistic front-electrode materials

We would now like to calculate the optimal TPV efficiency that should be achievable using front electrodes made of real materials, whose conductivity can be tuned via doping.

**Figure 4:**
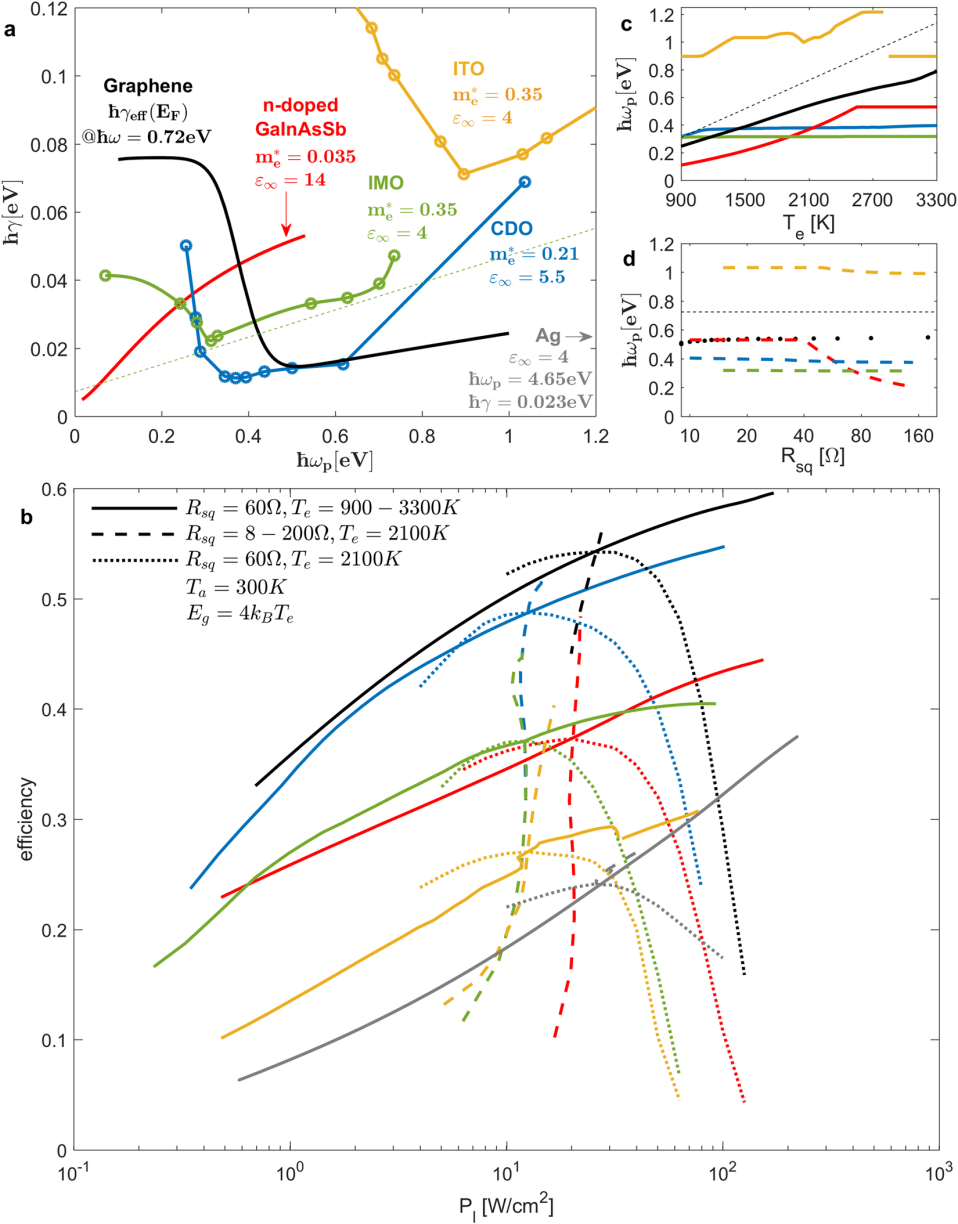
(**a**) Scattering loss rates several candidate electrode materials vs doping level (*E*_*F*_ for graphene), with shown the *ε*_∞_ and m* used to convert mobility to loss rate (see details in Methods); dashed line represents the model material of Figs 2 and 3. (**b**) Optimized efficiency vs load power per surface area *P*_*l*_ for the structure of Fig. 1 with the electrode materials of (**a**). (**c**) Optimal doping level (*E*_*F*_ for graphene) vs emitter temperature *T*_*e*_; dashed line is the semiconductor bandgap *E*_*g*_. (**d**) Optimal doping level (*E*_*F*_ for graphene) vs square resistance *R*_*sq*_; dashed line is the semiconductor bandgap *E*_*g*_.

A common method to create a tunable front electrode is to highly dope a front region of the same semiconductor thin film that also performs the photo-current generation. This process is often called “emitter diffusion”. Since we are examining the case of an ultra-thin single-mode film and trying to attain a relatively-low-resistance electrode by doping only a portion of this film, we assume (and confirm in simulations later, in Fig. 4c,d) that doping has to be so high that the semiconductor is degenerate in this electrode region. Therefore, minority-carrier recombination is really fast and the diffusion length therein is really short, so this degenerate-semiconductor electrode region does not contribute to photo-current or voltage and acts simply as a ‘plasmonic’ material with tunably-many free (majority) carriers. Assuming some Ga_x_In_1-x_As_y_Sb_1-y_ (GIAS) quaternary direct-gap semiconductor, a typical^15^ dependence of its electron mobility *μ* on the tunable doping level *N* is plotted in Fig. 4a, in terms of the equivalent functional *γ*(*ω*_*p*_), and is characterized by the same scattering mechanisms as those discussed earlier for our model electrode material.

Another class of tunable conductors highly-relevant for PV electrodes is that of conducting oxides. Their performance depends on many factors, such as constitutive oxide and dopant materials, dopant concentration, film thickness, deposition method, substrate material and temperature, annealing conditions and more. As is the case for all doped semiconductors, their limiting mechanism at high carrier concentrations is again ionized impurity scattering. However, the mobility of conducting oxides is often limited also at low doping by phenomena, such as low hopping/percolation ability, grain boundary scattering etc (see e.g. refs^16,17^). Therefore, conducting oxides often perform best at some optimal doping range. A commonly used oxide is Indium Oxide. When doped with Tin, Indium Tin Oxide (ITO) can become conductive with a plasma frequency below the visible region and with small loss rate, therefore it is the material of choice for front electrodes of many solar PV cells and LCD panels^13^. Based on some good-performance experimental demonstrations^17,18^, a typical ITO electron scattering rate versus doping *γ*(*ω*_*p*_) is plotted in Fig. 4a. It can be seen that the usable low-loss doping range of ITO is around its loss minimum at ${\omega }_{p}\approx 0.9\,eV\iff N\approx 8.15\cdot {10}^{20}\,c{m}^{-3}$, higher than typical TPV semiconductor bandgaps, so in most cases an ITO front electrode will be opaque at *ω*_*g*_. For this reason, in one previous investigation with a bulk PV cell^19^, only a 5 *nm*-thick 410Ω-resistance ITO electrode was shown to work without a detrimental effect to efficiency. Here, we will show that, by appropriately tuning the doping and in a thin-film-PV-cell geometry, lower-*R*_*sq*_ ITO electrodes can also be efficiently implemented. For a transparent TPV electrode, we need a plasma frequency deeper in the infrared. It turns out that doping Indium Oxide with Molybdenum (IMO) creates carriers with much higher mobility and that IMO can be doped to be transparent and low-loss in the infrared^20–22^. Moreover, another oxide, Cadmium Oxide doped with Dysprosium (CDO), was recently discovered to have even higher mobility^23^ and can therefore be really promising for infrared applications. Typical *γ*(*ω*_*p*_) curves for these oxides, shown in Fig. 4a, indicate that their optimal operating range, around ${\omega }_{p}\approx 0.31\,eV\iff N\approx {10}^{20}\,c{m}^{-3}$ for IMO and ${\omega }_{p}\approx 0.37\,eV\iff N\approx 1.15\cdot {10}^{20}\,c{m}^{-3}$ for CDO, is indeed applicable to TPV cells.

An upcoming very promising alternative for conducting electrodes is graphene, due to its extremely high carrier mobility. This two-dimensional material has been studied previously as electrode for bulk TPV cells^24,25^ and here we examine it for ultra-thin silver-backed cells. We consider *M* graphene monolayers, doped at a Fermi level *E*_*F*_, whose carrier mobility due to acoustic-phonon scattering is taken to scale with carrier density as *μ* = *A*/*N* ^2*D*^, where *A* constant (see Methods). With this scaling, the DC conductivity is constant, independent of carrier density, therefore the electrode square resistance is determined only by the number of monolayers (and can thus take only discrete values):

4 $${R}_{sq}=\frac{1}{{\sigma }_{DC}^{2D}M}=\frac{1}{q\mu {N}^{2D}M}=\frac{1}{qAM}\iff M=\frac{1}{qA{R}_{sq}}$$

We define via Eq. (3) an effective frequency-dependent scattering loss rate *γ*_*eff*_(*ω*,*E*_*F*_) for graphene, including all absorption mechanisms, and we plot it in Fig. 4a for some frequency *ω*. Like for conducting oxides, graphene also has an optimal doping range; in this case, though, it varies with the desired operating frequency *ω*, since the onset of inter-band absorption is roughly *E*_*F*_ < *ω*/2.

Finally, we also consider the case of using an extremely thin layer of silver as an electrode. Its doping is not tunable and it is opaque at infrared, but it is the lowest-loss plasmonic material (in terms of *γ*/*ω*_*p*_). Note, however, that at such ultra-thin scales, its loss rate does increase as the film thickness decreases^26^ (see Methods).

To test the performance of all these front-electrode material candidates, we performed 3 optimizations for each material on the structure of Fig. 1: one at *T*_*e*_ = 2100 °*K* and *R*_*sq*_ = 60Ω with varying output power per surface area *P*_*l*_ as a constraint, whose results always exhibit a peak efficiency at some power value, and then one at *T*_*e*_ = 2100 °*K* with varying *R*_*sq*_ and one at *R*_*sq*_ = 60Ω with varying *T*_*e*_, whose results follow the mentioned peaks with respect to the varying parameter. We note again that the material mobilities used in our simulations (and shown in Fig. 4a) are based on measurements of specific experimental configurations, so we consider them only as representative values for their respective materials over the entire parameter space we explore. The results are shown in Fig. 4b for the efficiency, in Fig. 4c,d for the optimal doping level, and in the “Supplementary Information” Fig. S4 for all optimal parameters.

The optimized efficiency curves in Fig. 4b suggest that, in comparing the different front-electrode material candidates, their performance unsurprisingly follows their loss ordering indicated in Fig. 4a. Graphene is the best material, followed closely by CDO; then IMO and GIAS are 10–15% worse in efficiency, while the opaque electrodes ITO and Ag are the least efficient, although efficient enough to be usable and certainly with better TPV performance than has ever been shown for these materials at practical electrode-resistance values. Fig. 4c confirms the design predictions we made earlier: As *T*_*e*_ varies, IMO, CDO and graphene choose a doping level close to their loss-rate minimum (except for CDO at low *T*_*e*_, which tends to smaller *ω*_*p*_ < *ω*_*g*_ for transparency), so their electrode thickness stays constant. GIAS at high *T*_*e*_ clamps at the highest possible doping level, which decreases with lower *T*_*e*_, but never to less than degenerate levels, confirming the analysis of our model material that the thickness of the electrode also affects the photonic design and cannot be too large. ITO shows the most interesting behavior, by being opaque at low *T*_*e*_ (and thus *ω*_*g*_) and close to its loss-rate minimum, but jumping to a transparent doping value at very high bandgaps, thus avoiding the lossy triple resonance; perhaps, for *T*_*e*_ around 2800 °*K*, a bandgap that deviates from the rule *E*_*g*_ = 4 *k*_*B*_ *T*_*e*_ might be better for efficiency. Fig. 4d suggests that doping levels do not change much from their optimal values as *R*_*sq*_ decreases, rather a thicker electrode is better (Fig. S4d). Therefore, the vacuum gap has to be reduced (Fig. S4b) to maintain impedance matching between the emitter and absorber modes, so the output power stays fairly constant although efficiency decreases (Fig. 4b), with the extra emitted power absorbed primarily inside the front electrode. Lastly, an interesting result is that the efficiency of an ultra-thin silver electrode is fairly insensitive to *R*_*sq*_, since a thicker film is less lossy and vice versa.

## Discussion

In this article, we focused on ultra-thin single-mode PV cells, motivated by the design principles that led to optimal efficiencies in our previous study^11^ and by the need to reduce the required amount of the expensive direct-bandgap semiconductor material. When this constraint is relaxed and we allow for multimode cells, other phenomena come into play. As the thickness of the semiconductor thin film increases, likely not all of it will be depleted (see Methods), so some bulk recombination inside it is expected. In the case of transparent-conducting-oxide and graphene front electrodes, we found via simulations (“Supplementary Information” Fig. S5) that, even assuming full depletion and ignoring this recombination, indeed a single-mode design is usually the most efficient. On the other hand, in the case of a diffused-emitter semiconductor electrode, a thicker electrode region can be used with lower doping and lower loss, so a multimode design could perhaps be more efficient. However, the doping cannot be lower than the one in the depletion region, necessary to attain the desired built-in voltage *V*_*bi*_ (see Methods). Moreover, absorber photonic states are now available below the bandgap for the emitter to emit into. Therefore, a more precise modeling of the electron distribution and bulk recombination in the cell is needed, and it is not straightforward whether one gains in efficiency or not.

Another important remark is that the cells recommended here are so thin that care must be taken to not exceed the breakdown field of the semiconductor. This is indeed the case for most designs shown here, but the values are often within an order of magnitude from the limit.

In conclusion, we have carefully analyzed, for the first time to our knowledge, the problem of designing conducting electrodes for high-efficiency near-field thin-film PV cells. The design principles suggested here are not only applicable to incoming thermal near-infrared radiation in a near-field TPV system but also to any other source of near-field radiation of any frequency. We identified and compared real materials who appear to be good candidates for near-field TPV cells, and demonstrated potential performances: for example, at *T*_*e*_ = 2100 °*K* and *R*_*sq*_ = 60Ω, using a TiC emitter and a GaSb absorber, then one could achieve ~12 *W*/*cm* ^2^ load power with ~48% efficiency with a 10 *nm*-thick CDO electrode, 310 *nm* vacuum gap and 63 *nm*-thick absorber, or ~12 *W*/*cm* ^2^ load power with ~37% efficiency with a 40 *nm*-thick IMO electrode, 280 *nm* vacuum gap and 58 *nm*-thick absorber, or ~27 *W*/*cm* ^2^ load power with ~24% efficiency with a 1.1 *nm*-thick Silver electrode, 110 *nm* vacuum gap and 90 *nm*-thick absorber. This performance could be very promising for efficient high-power TPV devices, once practical problems (maintaining small gaps and large temperature differentials^27^) are resolved.

## Methods

### Calculational approach

Our calculations for the planar TPV systems under examination were performed using the formulas

5 $$\begin{array}{rcl}{P}_{e}(V) & = & {\int }_{0}^{\infty }\frac{d\omega }{2\pi }\hslash \omega \,{\int }_{0}^{\infty }\frac{{k}_{xy}d{k}_{xy}}{2\pi }\,\{[{{\rm{\Theta }}}_{0{T}_{e}}(\omega )-{{\rm{\Theta }}}_{0{T}_{a}}(\omega )]\,{\epsilon }_{e}\,(\omega ,{k}_{xy})\\  &  & -\,[{{\rm{\Theta }}}_{0{T}_{a}}(\omega )-{{\rm{\Theta }}}_{V{T}_{a}}(\omega )]\,{\epsilon }_{eg}\,(\omega ,{k}_{xy})\}\end{array}$$

6 $$\begin{array}{rcl}I(V) & = & {\int }_{0}^{\infty }\frac{d\omega }{2\pi }q\,{\int }_{0}^{\infty }\frac{{k}_{xy}d{k}_{xy}}{2\pi }\,\{[{{\rm{\Theta }}}_{0{T}_{e}}(\omega )-{{\rm{\Theta }}}_{0{T}_{a}}(\omega )]\,{\epsilon }_{eg}\,(\omega ,{k}_{xy})\\  &  & -\,[{{\rm{\Theta }}}_{0{T}_{a}}(\omega )-{{\rm{\Theta }}}_{V{T}_{a}}(\omega )]\,{\epsilon }_{g}\,(\omega ,{k}_{xy})\}\end{array}$$

where *ω* is the angular frequency, *k*_*xy*_ is the in-plane wavevector, ${\epsilon }_{ij}(\omega ,{k}_{xy})$ is the *thermal transmissivity* from *j* to *i*, ${\epsilon }_{j}(\omega ,{k}_{xy})={\sum }_{i\ne j}{\epsilon }_{ij}(\omega ,{k}_{xy})$ the *thermal emissivity* of *j*, ${{\rm{\Theta }}}_{{V}_{j}{T}_{j}}(\omega )=1/\{\exp [(\hslash \omega -q{V}_{j})/{k}_{B}{T}_{j}]-1\}$ the Planck distribution for the mean number of photons at voltage *V*_*j*_ and temperature *T*_*j*_, *P*_*e*_ the power *per surface area* emitted by the emitter *e*, *V* the voltage across the semiconductor depletion region and the load, *I* the current *per area* output to the load (leading to output load power *per area* ${P}_{l}=V\cdot I$), and *g* signifies the voltage-generating inter-band absorption mechanism associated with the semiconductor electronic band*g*ap. The calculation of thermal transmissivities/emissivities involves, for planar layered systems, an exact semi-analytical scattering-matrix method^11,28^. The integrals of Eqs (5) and (6) were calculated numerically over a dense non-uniform *ω* − *k*_*xy*_ grid, with frequency and wavevector cutoffs 3*ω*_*g*_ and *π*/(minimum layer thickness) respectively, for the Planck Θ factors and all $\epsilon$ factors to have sufficiently decayed to zero (see also next paragraph). Extensive details for the definition and calculation of thermal transmissivities/emissivities and for the derivation of Eqs (5) and (6) are given in the “Supplementary Information” of ref.^11^.

The ${\epsilon }_{g}$ term in Eq. (6) represents a first-principles calculation of the radiative recombination in the semiconductor film under a voltage (or chemical potential) *V* and thereby of the associated recombination current of the PV diode. Some of this recombinative radiation may be absorbed inside the film (assumed zero in this article), and the rest exits the film and is absorbed in the losses of surrounding layers, which have zero chemical potential (thus the driving term ${{\rm{\Theta }}}_{0{T}_{a}}-{{\rm{\Theta }}}_{V{T}_{a}}$ in Eq. (6) is nonzero). However, the radiative flux between two planar layers scales as ~1/distance^2^ in the absence of spatial dispersion^29^. Therefore, if the semiconductor and the lossy electrode layers were absolutely adjacent (touching), then this radiative flux would numerically diverge, leading to infinite recombination current: essentially ${\epsilon }_{g}(\omega ,{k}_{xy})$ for TM polarization would be finite ($0 < {\epsilon }_{g} < 1$) as *k*_*xy*_ → ∞ at some *ω*, so its *k*_*xy*_-integral would diverge. To avoid this divergence, we insert two lossless dielectric (*ε* = 4) extremely-thin ‘convergence layers’ of thicknesses *d*_*front*_ and *d*_*back*_ (yellow layers in Fig. 1), so ${\epsilon }_{g}$ has sufficiently decayed to zero by *k*_*xy*_ = *π*/min(*d*_*front*_, *d*_*back*_). In a real structure, these layers can be ‘window layers’ to passivate the interfaces and reduce surface-recombination velocities.

We believe that this purely-photonic first-principles calculational approach has the potential to capture more accurately the physical *dependence* of the radiative recombination rate on the system’s ultra-thin resonant geometry compared to the common method of citing a constant rate, experimentally measured for the same semiconductor material but *some other* system geometry. Surface and non-radiative recombination mechanisms could be accounted for by an external additive term in Eq. (6), but they are assumed negligible: III-V direct-bandgap semiconductor ultra-thin-film PV cells can be grown epitaxially such that very low defect-density is achieved, and thus surface recombination can, in principle, be practically eliminated via the use of high-quality passivation layers, and non-radiative recombination mechanisms (e.g. Auger, Shockley-Hall-Read) are, in fact, insignificant compared to radiative recombination (see analysis in “Supplementary Information” and refs^10,30,31^). On the other hand, with our method, substantial simplifications on the electronic details of the pn-junction are made. For example, in our model, the chemical potential (quasi-Fermi-level separation) changes abruptly from *V* (constant throughout the depleted semiconductor-absorber film) to 0 (in the ‘convergence layers’), which is not strictly correct. Furthermore, the doping of the semiconductor has to be high enough that the built-in voltage^32^ ${V}_{bi}={k}_{B}{T}_{a}/q\cdot ln({N}_{D}{N}_{A}/{N}_{i}^{2})$, where *N*_*D*_, *N*_*A*_, *N*_*i*_ the donor, acceptor and intrinsic concentrations respectively, is larger than the desired optimal operating voltage *V*; but then the depletion-region width^32^ $W=\sqrt{2{\varepsilon }_{a}/q(1/{N}_{D}+1/{N}_{A})\,({V}_{bi}-V-2{k}_{B}{T}_{a}/q)}$ turns out to be itself so small, that, in reality, it may not extend throughout even an ultra-thin single-mode semiconductor film. In short, our model does not predict the precise distributions of carriers and Fermi levels with depth, however, we do not expect the efficiency error to be significant (say <3%) in most cases (see analysis in “Supplementary Information” and Fig. S6). Overall, no existing method is perfect, but a key message of this article is that near-field TPV performance in all cases is heavily affected by the photonic design and losses, which we calculate here with great precision. Therefore, we believe that our calculational method models near-field ultra-thin-film TPV systems adequately enough to provide a good sense of potential performance. Extensions of the model should include electrical losses of the electrodes and power required to cool the PV cell and maintain it at room temperature.

For all results presented, the structures have been optimized to maximize the TPV efficiency *η* = *P*_*l*_/*P*_*e*_, so that fair comparisons among optimal systems can be made. The optimization parameters are the emitter plasma-frequency *ω*_*p*,*e*_, the vacuum-gap width *d*_*v*_, the front-electrode doping level *ω*_*p*_ (or *E*_*F*_ for graphene layers, except for silver and for Fig. 2), the semiconductor-absorber thickness *d*_*a*_, the ‘convergence layers’ thicknesses *d*_*front*_, *d*_*back*_, and the load voltage *V*. The electrode thickness *d* is calculated via Eq. (2), the single-mode condition is imposed via a maximum limit on *d*_*a*_, the near-field (high-power) regime is maintained via a maximum limit on *d*_*v*_, and *d*_*front*_, *d*_*back*_ are limited in the range (0.001–0.005)*λ*_*g*_. The efficiency definition does not include electrical losses due to (series Δ*η*/*η* and shunt) electrode resistances, although the square resistance *R*_*sq*_ of the front electrode is always specified (to determine its necessary thickness *d* via Eq. (2)) and its associated photonic losses are calculated. The colored curves of Fig. 3 and the dotted curves of Fig. 4 were obtained using constrained optimization, with output power per area *P*_*l*_ as a constraint. Again, in all cases, the semiconductor bandgap was chosen as *E*_*g*_ = 4 *k*_*B*_ *T*_*e*_.

### Materials modeling

We model free carriers, in the plasmonic material and in the PV-cell back and front electrodes via the Drude model, namely the relative dielectric permittivity

7 $$\varepsilon (\omega )={\varepsilon }_{\infty }(1-\frac{{\omega }_{p}^{2}}{{\omega }^{2}+{\rm{i}}\gamma \omega })$$

with plasma frequency ${\omega }_{p}=q\sqrt{N/{\varepsilon }_{o}{\varepsilon }_{\infty }{m}^{\ast }}$ and loss factor *γ* = *q*/μm*, where *N* is the carrier density, m* the effective mass of the carriers (electrons or holes) and *μ*(*N*) the population-dependent carrier mobility.

For the plasmonic emitter, we leave *ω*_*p*,*e*_ as an optimization-design variable, assume *ε*_∞,*e*_ = 1 (for simplicity) and estimate the temperature-dependent loss factor as^11,33^ *γ*_*e*_(*T*) = *γ*_*lin*_(*T*)/(1 + *γ*_*lin*_(*T*)/*γ*_∞_) with *γ*_*lin*_(*T*) = *γ*_*o*_(1 + *αT*), *α* = 0.002/°*K*, *γ*_*o*_ = 0.05*ω*_*p*,*e*_ and *γ*_∞_ = 2*γ*_*o*_.

The back-electrode silver is modeled with *ε*_∞,*Ag*_ = 4, *ħω*_*p*,*Ag*_ = 4.65 *eV* and *ħγ*_*Ag*_ = 0.023 *eV* ^26,34,35^.

The front-electrode materials’ parameters are shown in Fig. 4a, where the mobility-vs-carrier dependence *μ*(*N*) was converted to the form *γ*(*ω*_*p*_) using the shown corresponding values of *ε*_∞_ and m*. Specifically, for degenerate Ga_x_In_1−x_As_y_Sb_1−y_ semiconductors, we used *ε*_∞_ = 14, electron effective mass ${m}_{e}^{\ast }=0.035\,{m}_{e}$ and electron mobility ${\mu }_{e}({N}_{D})=420+8500/[1+{({N}_{D}/5\times {10}^{17}c{m}^{-3})}^{0.7}]\,c{m}^{2}/V\,sec$ ^15^, and limited the maximum achievable doping level to ${N}_{D}={10}^{20}\,c{m}^{-3}\Rightarrow \hslash {\omega }_{p}=0.53\,eV$ ^36^. Moreover, since minority carriers are expected to recombine really fast in this degenerate electrode, as discussed in the main text, we ignored the photo-current-generating inter-band-absorption term in the semiconductor permittivity.

In the case of conducting oxides, we extracted and interpolated the experimental mobility data *μ*(*N*) of ITO from refs^17,18^, of IMO from refs^21,22^ and of CDO from ref.^23^, while we used *ε*_∞_ = 4, m* = 0.35 *m*_*e*_ for Indium Oxide^37^ and *ε*_∞_ = 5.5, m* = 0.21 *m*_*e*_ for Cadmium Oxide (see Supplemental Material in ref.^23^). Again, the mobility of these oxides depends on multiple fabrication parameters (such as film thickness), so, using mobility values taken from these individual experimental demonstrations, will give us only an indicative performance of these materials.

The *M* graphene monolayers were spaced by 1 *nm*-thick dielectric (*ε* = 4) films and each graphene layer was modelled via its 2D conductivity, which has both an intra-band and an inter-band term^38^:

8 $$\begin{array}{rcl}{\sigma }_{gr}^{2D}(\omega ) & = & \frac{{q}^{2}}{\pi \hslash }\frac{\frac{2{k}_{B}{T}_{a}}{\hslash }\,\mathrm{ln}\,(2\,\cosh \,\frac{{E}_{F}}{2{k}_{B}{T}_{a}})}{\gamma -{\rm{i}}\omega }\\  &  & +\frac{{q}^{2}}{4\hslash }\,[G(\omega /2)+\frac{\omega }{{\rm{i}}\pi }\,{\int }_{0}^{\infty }du\,\frac{G(u)-G(\omega /2)}{{u}^{2}-{(\omega /2)}^{2}}]\end{array}$$

where *T*_*a*_ = 300 °*K* the absorber (PV cell) temperature, ${E}_{F}=\hslash {v}_{gr}\sqrt{\pi {N}^{2D}}$ the Fermi level due to carriers of density *N* ^2*D*^ and velocity *v*_*gr*_ = 10^6^ *m*/*sec*, and *G*(*u*) = sinh(*ħu*/*k*_*B*_ *T*_*a*_)/[cosh(*E*_*F*_/*k*_*B*_ *T*_*a*_) + cosh(*ħu*/*k*_*B*_ *T*_*a*_)]. The loss rate *γ* consists of two terms, due to scattering of free carriers with acoustic and optical phonons:

9 $$\gamma ={\gamma }^{AP}+{\gamma }^{OP}(\omega )\approx \frac{q{v}_{gr}^{2}}{\mu {E}_{F}}+0.04\sqrt{\frac{{E}_{F}}{\hslash }(\omega -{\omega }^{OP})}$$

The carrier mobility due to acoustic-phonon scattering is taken to scale as^39–41^ *μ* = *A*/*N* ^2*D*^, using *A* = 3.47 × 10^16^/*Vsec*, appropriate^40^ for room temperature and the very high doping levels (${N}^{2D} > {10}^{13}\,c{m}^{-2}\Rightarrow {E}_{F} > 0.37\,eV$) which will be required here (see Fig. 4c). (Note that refs^24,25,42^ used a constant *γ* ^*AP*^, which would imply $\mu \sim 1/\sqrt{{N}^{2D}}$.) *ħω* ^*OP*^ = 0.2 *eV* is the optical phonon frequency in graphene and the second term in Eq. (9) is a very rough (and likely pessimistic) approximation, based on Fig. 4 of ref.^42^, of the optical-phonon-related scattering rate *γ* ^*OP*^, which is dependent on the frequency *ω* of the photon.

For ultra-thin-film silver front electrodes, we account for the fact that, when the film thickness becomes smaller than the mean electron free path, the electron collisions with the silver boundaries affect the scattering rate scaling as^26^ *ħγ* = 0.023 *eV* + 0.25 *v*_*Ag*_/*d*, where ${v}_{Ag}=1.39\cdot {10}^{6}\,m/sec$. The silver film thickness *d* required to get a certain electrode *R*_*sq*_ can still be found from Eq. (2), with *γ*(*d*) resulting in a second-order equation in *d*.

As stated in the main text, the depletion region is assumed to extend throughout the PV-cell semiconductor film, due to its tiny single-mode thickness. Therefore, the carrier density inside it will be negligibly low. Thus, assuming again some Ga_x_In_1-x_As_y_Sb_1-y_ quaternary, we model it using a dielectric permittivity accounting only for inter-band absorption across its bandgap *ω*_*g*_ (no free-carrier absorption Drude term)^11^.

10 $${\varepsilon }_{a}(\omega )=14+{\rm{i}}0.7({\omega }_{g}/\omega )\sqrt{14(\omega /{\omega }_{g}-1)}$$

Note that we have used two very different permittivity models for the same Ga_x_In_1−x_As_y_Sb_1−y_ semiconductor material, according to whether it is a degenerate front electrode or the depleted voltage-generating pn junction.

## Electronic supplementary material

SUPPLEMENTARY INFORMATION
