# Supplementary material for: Transparent and ‘opaque’ conducting electrodes for ultra-thin highly-efficient near-field thermophotovoltaic cells
Source: Sci Rep. 2017 Oct 25;7:14046. doi: 10.1038/s41598-017-13540-8 (PMC5656648; doi:10.1038/s41598-017-13540-8)
Supplement: Supplementary file 1 — SUPPLEMENTARY INFORMATION [file 41598_2017_13540_MOESM1_ESM.pdf]

Transparent and ‘opaque’ conducting electrodes for ultra-thin highly-efficient near-field thermophotovoltaic cells

Aristeidis Karalis[1] and J. D. Joannopoulos[1,2]

SUPPLEMENTARY INFORMATION

For completeness and to ensure reproducibility of our results, we provide Figures (S1-S4) for all the optimization parameters corresponding to the results of the Figures 2a, 3a, 3b and 4b.

We also show in Figure S5 some results which give insight about the relative performance of *not* ultra-thin PV cells, which support two photonic modes, and we also include a section (and Figure S6) to estimate the error of our simplified electronic modeling of the pn junction.

For convenience, we repeat here Figure 1 from the main text, so that the structural and material parameters can be seen.

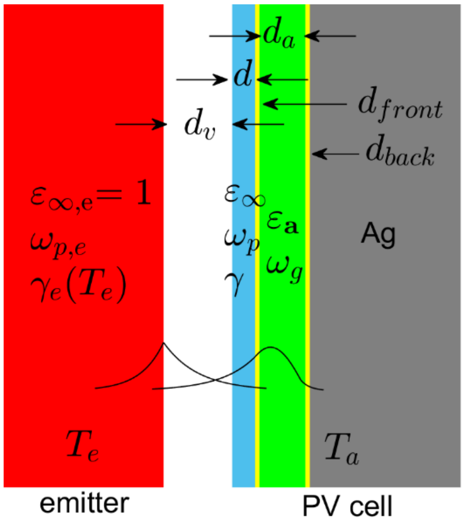

[1]Research Laboratory of Electronics, Massachusetts Institute of Technology, Cambridge, MA 02139, USA
[2]Department of Physics, Massachusetts Institute of Technology, Cambridge, MA 02139, USA
Correspondence: Aristeidis Karalis, Email: aristos@mit.edu, Phone: +1 (617) 253-6798

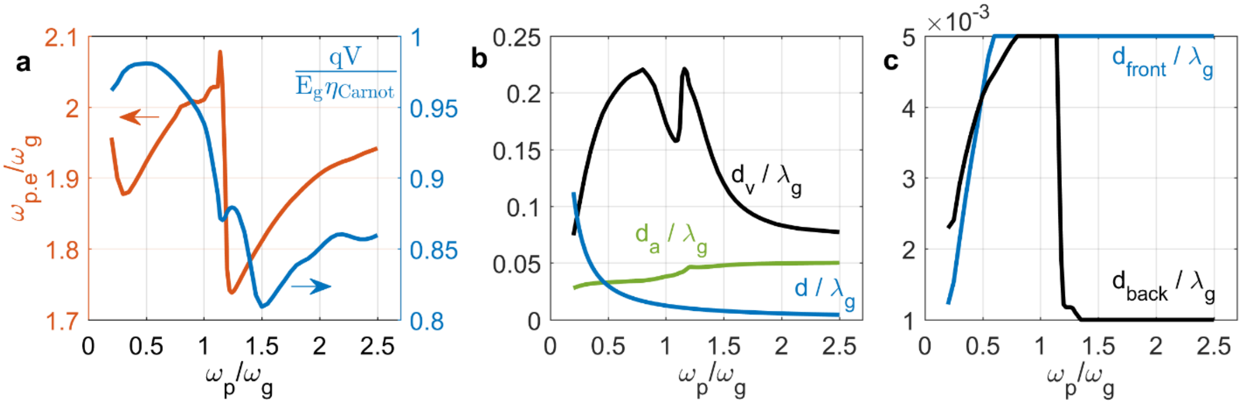

Supplementary Figure S1. Optimization parameters for results of Fig. 2a: **(a)** (left) Normalized emitter plasma frequency $\omega_{p,e}$ and (right) normalized load voltage V. **(b)** Normalized vacuum-gap width d_v, front-electrode thickness d and semiconductor-absorber thickness d_a. **(c)** Normalized front and back 'convergence layer' thicknesses d_{front} and d_{back}.

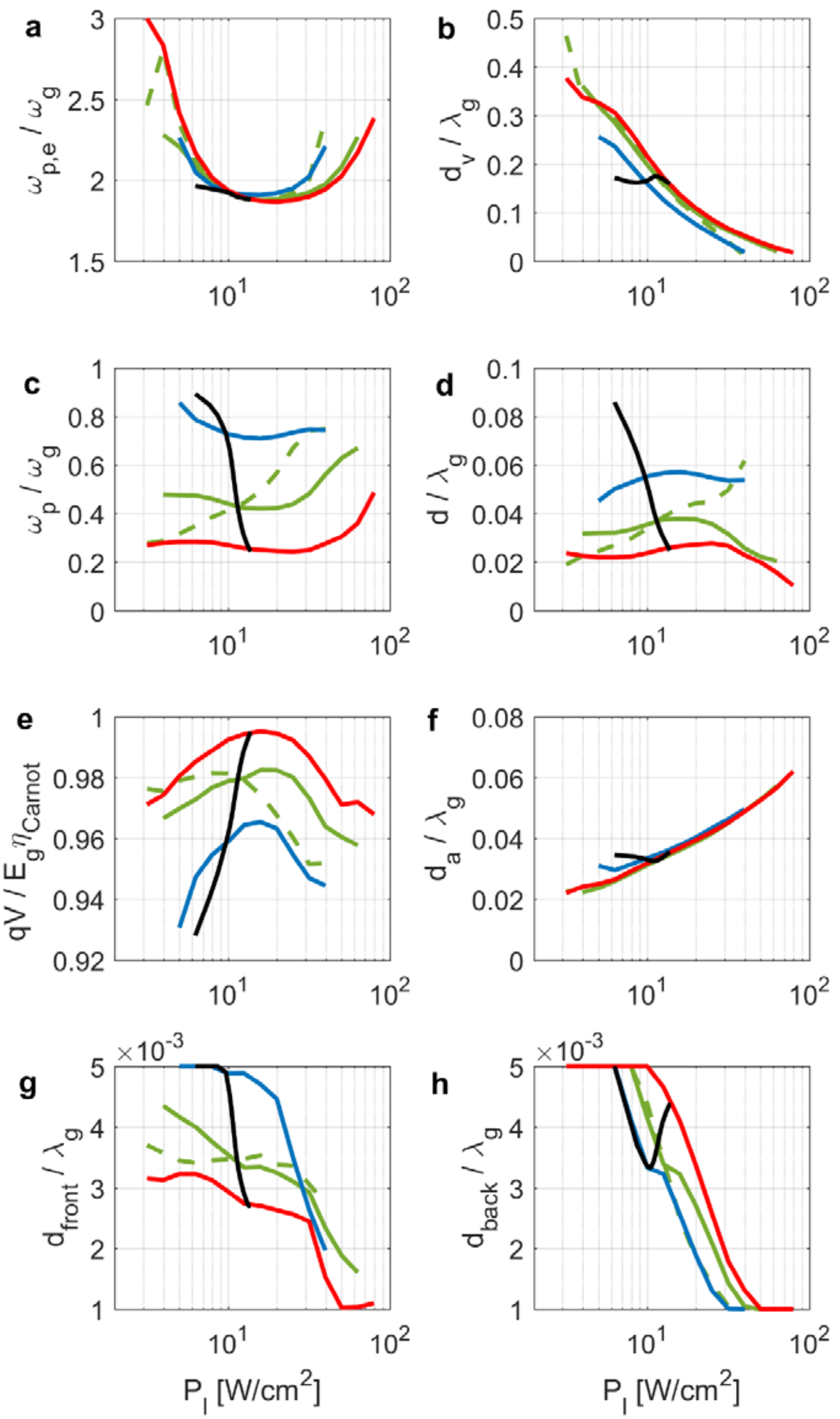

Supplementary Figure S2. Optimization parameters for results of Fig. 3a (with same color coding): **(a)** Normalized emitter plasma frequency $\omega_{p,e}$. **(b)** Normalized vacuum-gap width d_v. **(c)** Normalized front-electrode plasma frequency ω_p; note that optimal value is always $\omega_p < \omega_g$. **(b)** Normalized front-electrode thickness d. **(e)** Normalized load voltage V; note that optimal value is always close to $\hbar\omega_g\eta_{Carnot}/q$. **(f)** Normalized semiconductor-absorber thickness d_a; note that optimal value is independent of R_{sq}. **(g)** Normalized front 'convergence layer' thickness d_{front}. **(h)** Normalized back 'convergence layer' thickness d_{back}; note that the optimized efficiency is fairly insensitive to both 'convergence layers', so the convergence of Figs. (g,h) is not perfect.

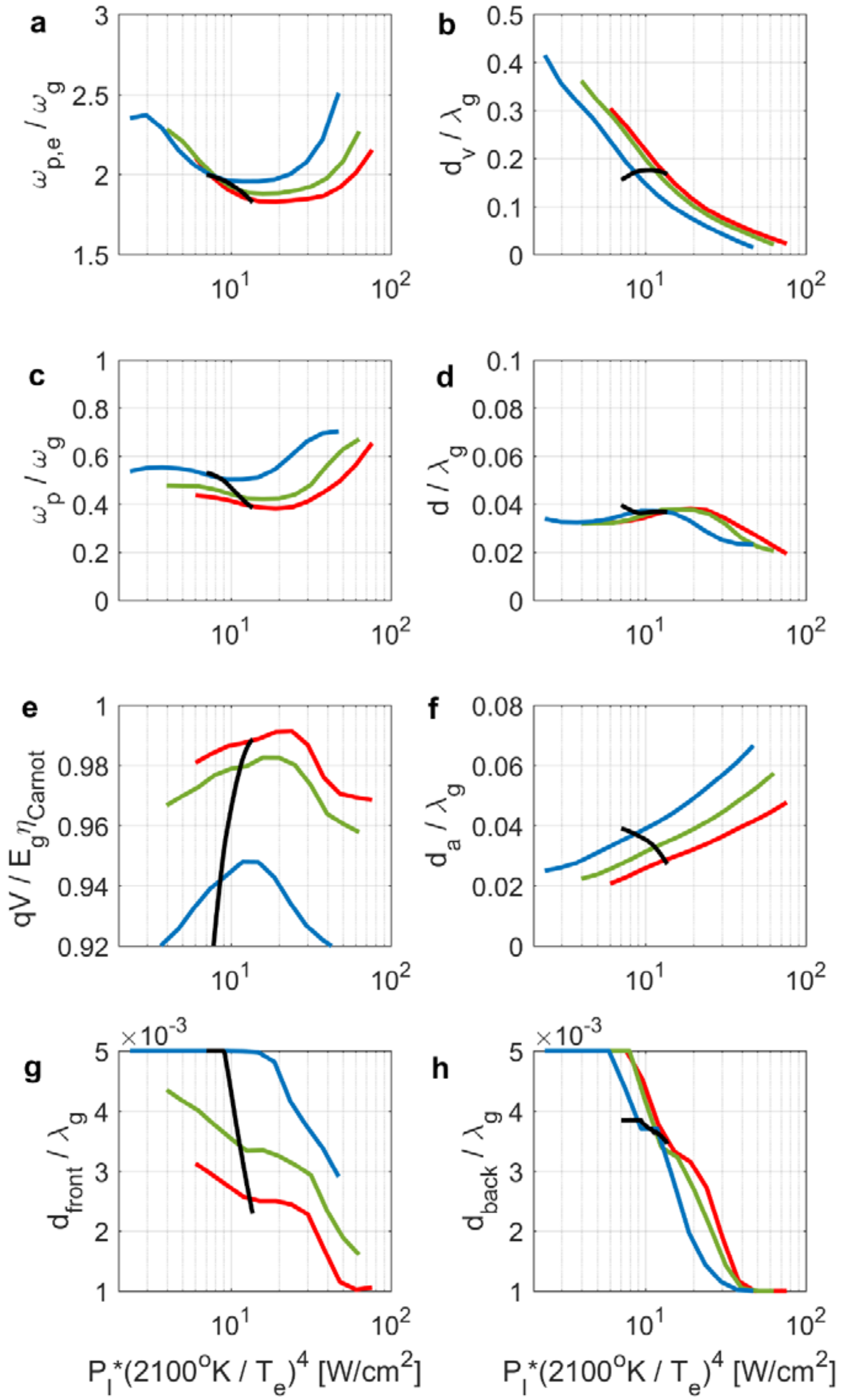

Supplementary Figure S3. Optimization parameters for results of Fig. 3b (with same color coding): **(a)** Normalized emitter plasma frequency $\omega_{p,e}$. **(b)** Normalized vacuum-gap width d_v. **(c)** Normalized front-electrode plasma frequency ω_p; note that optimal value is always $\omega_p < \omega_g$. **(b)** Normalized front-electrode thickness d. **(e)** Normalized load voltage V; note that optimal value is always close to $\hbar\omega_g \eta_{Carnot}/q$. **(f)** Normalized semiconductor-absorber thickness d_a. **(g)** Normalized front 'convergence layer' thickness d_{front}. **(h)** Normalized back 'convergence layer' thickness d_{back}; note that the optimized efficiency is fairly insensitive to both 'convergence layers', so the convergence of Figs. (g,h) is not perfect.

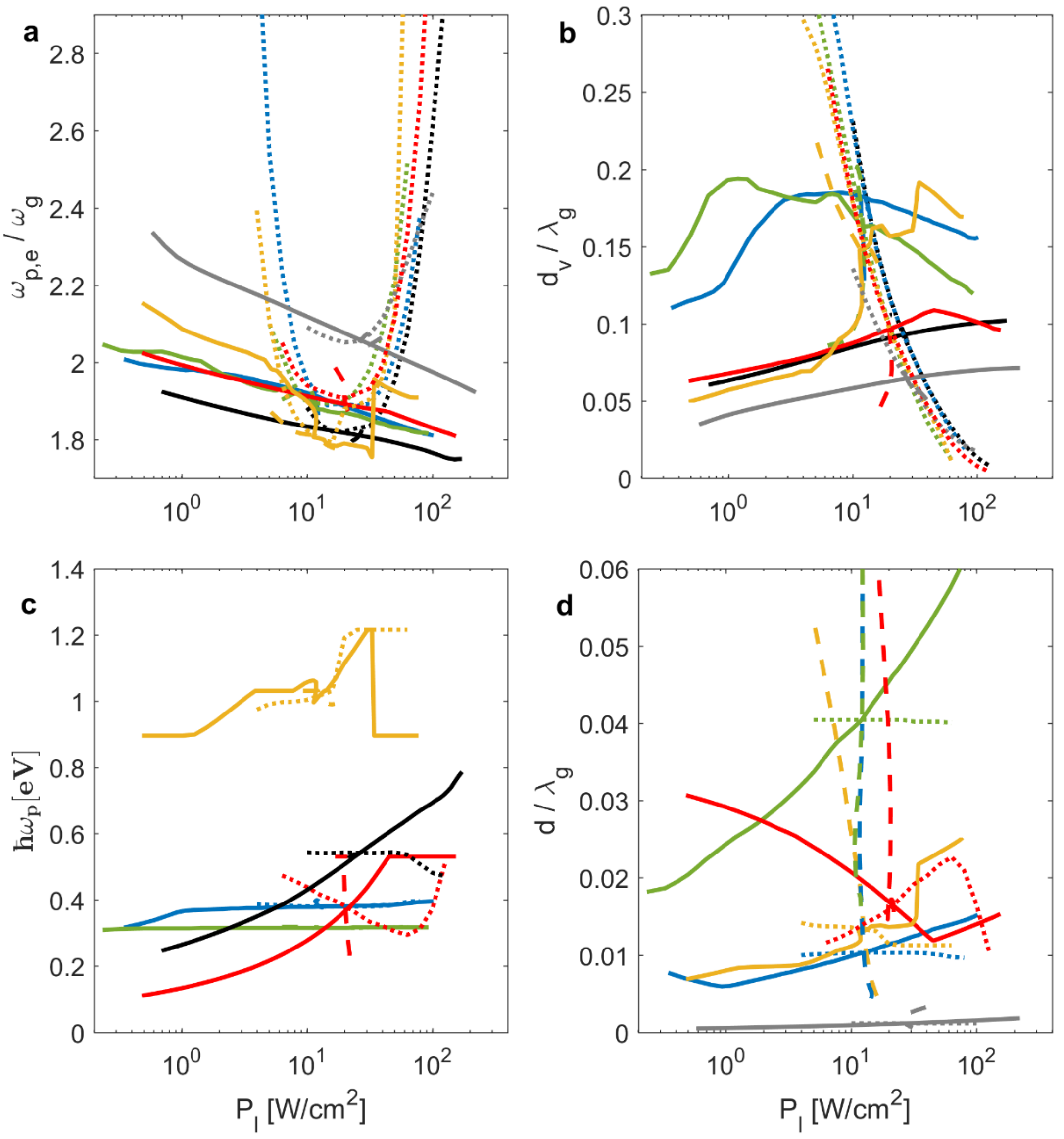

a
b
c
d
$\omega_{p,e} / \omega_g$
d_v / λ_g
$\hbar\omega_p [eV]$
d / λ_g
P_I [W/cm^2]
P_I [W/cm^2]

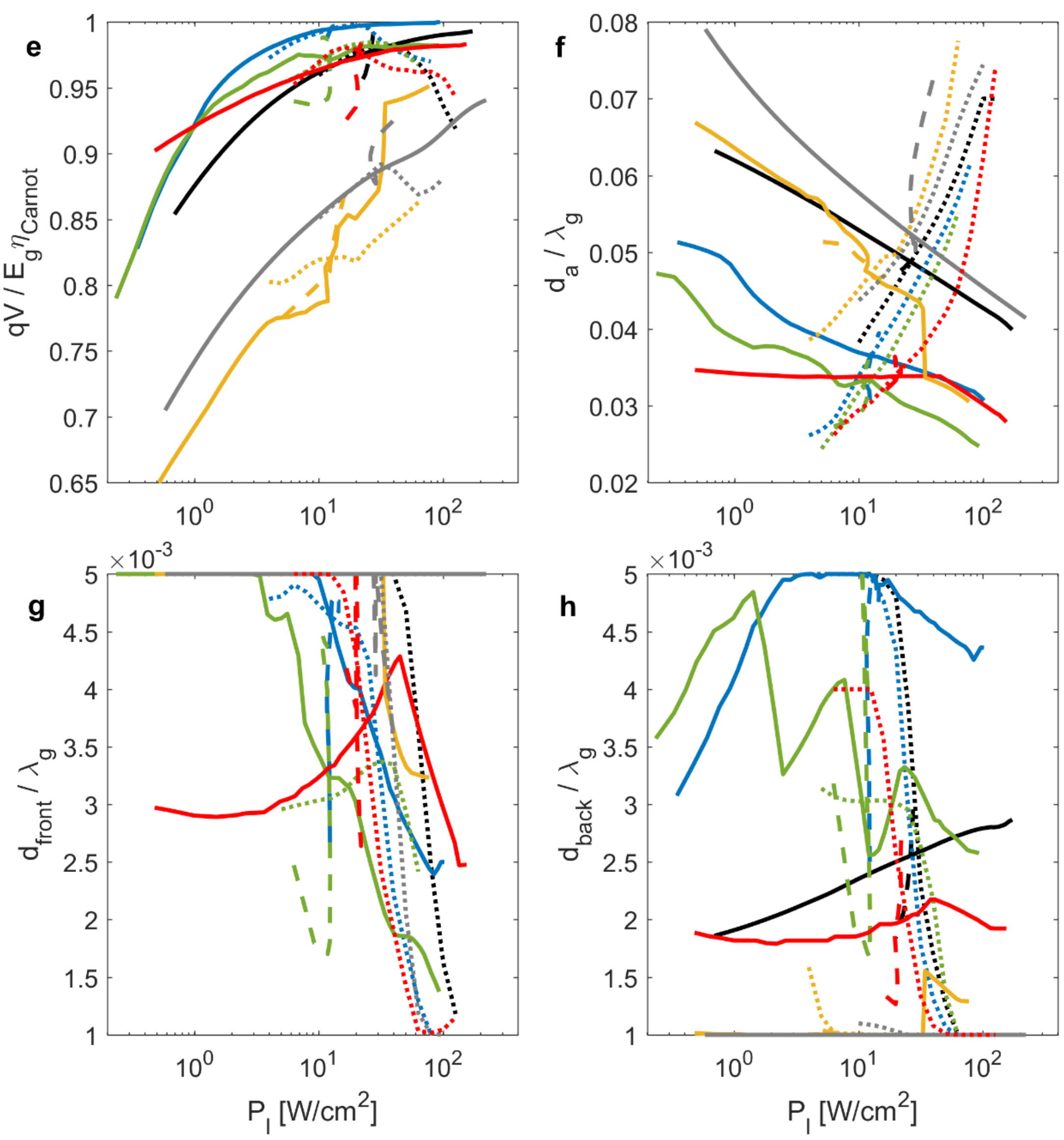

Supplementary Figure S4. Optimization parameters for results of Fig. 4b (with same color coding): **(a)** Normalized emitter plasma frequency $\omega_{p,e}$. **(b)** Normalized vacuum-gap width d_v. **(c)** Front-electrode plasma frequency ω_p. **(b)** Normalized front-electrode thickness d. **(e)** Normalized load voltage V. **(f)** Normalized semiconductor-absorber thickness d_a. **(g)** Normalized front 'convergence layer' thickness d_{front}. **(h)** Normalized back 'convergence layer' thickness d_{back}; note that the optimized efficiency is fairly insensitive to both 'convergence layers' in most cases, so the convergence of Figs. (g,h) is not perfect.

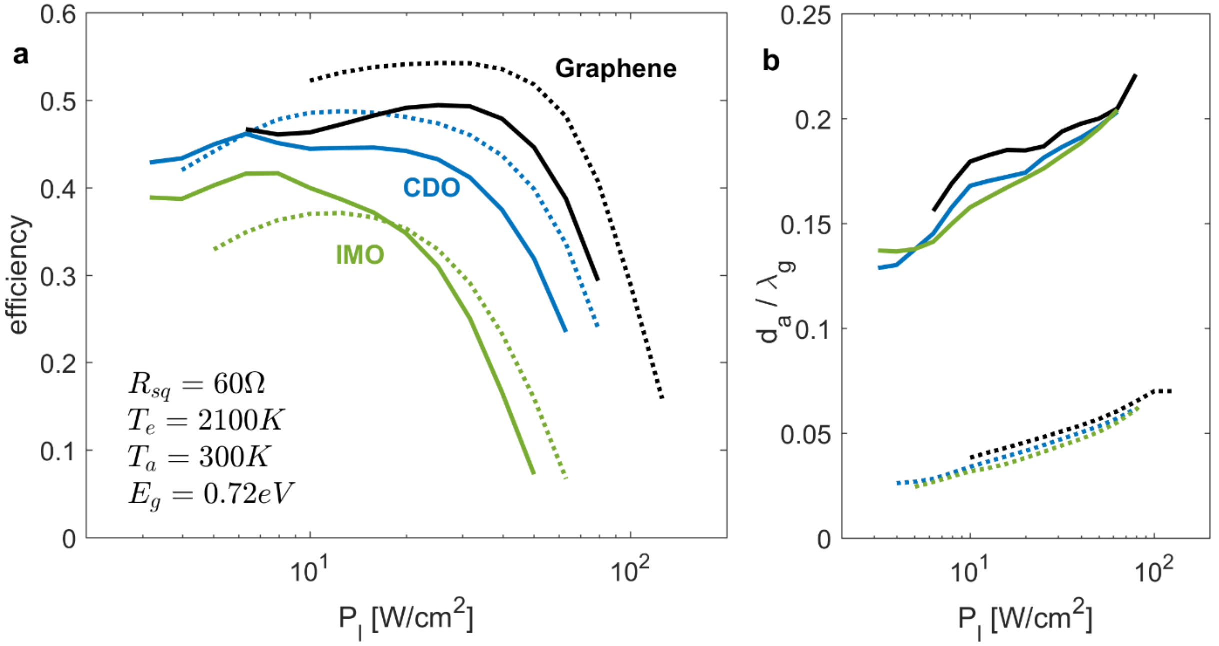

Supplementary Figure S5. Optimization results vs load power per surface area P_l for the structure of Fig.1 with some of the electrode materials of Fig.4a, with absorber designed to support a single mode (dotted curves, same as those in Figs.4b and S4f) and two modes (solid curves): **(a)** Efficiency η; single-mode design is more efficient in most cases, even after ignoring the radiative recombination inside the two-mode absorber. **(b)** Normalized semiconductor-absorber thickness d_a; two-mode absorber is considerably thicker (as expected), so the depletion region cannot extend throughout it.

Accuracy of simplified pn-junction electronic modeling

To estimate the order of magnitude of the error of our simplified pn-junction electronic modeling, we will use some standard pn-junction analytical formulas on the structure of Figure 2 at the highest-efficiency operating point $\omega_{p,1}$. The bandgap is $E_g = 0.72eV$ and the optimization results include $qV/E_g\eta_{\text{Carnot}} = 0.98 \Rightarrow V = 0.605V$ (see Figure S1a), $d_a/\lambda_g = 0.033 \Rightarrow d_a = 56.5nm$ (see Figure S1b) and $I_{dk} = 67pA/cm^2$, where I_{dk} is the 'dark' recombination current per surface area of the PV cell.

We will use material parameters for $Ga_xIn_{1-x}As_ySb_{1-y}$ semiconductors from Ref. 1. Therefore $N_C = 1.5 \cdot 10^{17}cm^{-3}$, $N_V = 7 \cdot 10^{18}cm^{-3}$ gives $n_i = \sqrt{N_C N_V}\exp(-E_g/2qV_a) = 8.5 \cdot 10^{11}cm^{-3}$, where $qV_a = k_B T_a = 25.8meV$ at $T_a = 300°K$.

We need high-enough doping that the internal-bias voltage V_{bi} exceeds the desired operating voltage V. Let us consider a highly asymmetric ('one-sided') junction with p[+] 'emitter' doping $N_A = 10^{19}cm^{-3}$ and n 'base' doping $N_D = 5 \cdot 10^{16}cm^{-3}$. Then [2] $V_{bi} = V_a \cdot ln(N_D N_A/n_i^2) = 0.705V$ and the depletion region has roughly a width[2] $W = \sqrt{2\varepsilon_a/q(1/N_D + 1/N_A)(V_{bi} - V - 2V_a)} = 38.6nm$ (using $\varepsilon_a = 14$), so indeed it extends throughout almost the entire thickness d_a of the semiconductor absorber (in fact, it is $55.7nm$, if the correction term $-2V_a$ is omitted) and we can safely assume that the non-depleted part ($d_a - W = 17.9nm$) lies in the wider quasi-neutral n 'base'.

In the calculation of I_{dk} we assumed that it is entirely due to radiative recombination throughout the absorber, so we can extract the radiative-recombination coefficient B via $I_{dk} \approx qBn_i^2 d_a \Rightarrow B \approx 10^{-10}cm^3/s$, which agrees with theoretically[1] and experimentally[3] derived values for $Ga_xIn_{1-x}As_ySb_{1-y}$ semiconductors. Using mobilities from Ref. 1, in the p[+] region minority electrons have $\mu_e(N_A) = 420 + 8500/[1 + (N_A/5 \times 10^{17}cm^{-3})^{0.7}]\ cm^2/Vs = 1350cm^2/Vs$ and in the n region minority holes have $\mu_h(N_D) = 110 + 500/[1 + (N_D/9 \times 10^{17}cm^{-3})^{0.66}]\ cm^2/Vs = 545cm^2/Vs$, so the respective diffusion constants are $D_e = V_a\mu_e = 35cm^2/s$ and $D_h = V_a\mu_h = 14cm^2/s$. The radiative lifetimes are $\tau_e = 1/BN_A = 1ns$ and $\tau_h = 1/BN_D = 200ns$, so the corresponding diffusion lengths $L_e = \sqrt{D_e\tau_e} = 1.9\mu m$ and $L_h = \sqrt{D_h\tau_h} = 16.7\mu m$, which are much larger than the absorber thickness, the preferred scenario for a 'short' PV diode, so that bulk recombination in quasi-neutral regions is minimized[4]. Therefore, even if one were to calculate the precise solution (via the drift-diffusion equations) for the minority-carrier distributions and the recombination current, those should not depend on the diffusion lengths L_e and L_h, and the relevant dimensions shall be the absorber thickness d_a and depletion-region width W.

This will also hold for the recombination current due to non-radiative mechanisms. Considering that the n 'base' occupies almost entirely the absorber, we will conservatively (and for simplicity) assume that non-radiative recombination of minority holes occurs throughout the absorber d_a (namely also in the depletion region W). Thus we can get a rough estimate for the current due to each non-radiative mechanism[5]:

- Very low surface-recombination velocities down to $v_{sr} = 10cm/s$ (and below) have been experimentally demonstrated both for GaAs[6,7] and for black Si[8,9,10], so we can estimate surface-recombination current $I_{sr} = qn_i^2/N_D \cdot v_{sr} = 23pA/cm^2$.
- Very long Shockley-Read-Hall (SRH) recombination lifetimes of around $\tau_{SRH} = 1\mu s$ have been measured experimentally[3,11,12,6] and even larger are estimated[13] for record-efficiency GaAs solar cells[14], so we can estimate SRH-recombination current $I_{SRH} = qn_i^2/N_D \cdot d_a/\tau_{SRH} = 13pA/cm^2$.

- The Auger recombination coefficient has been determined[1] approximately $C = 2 \cdot 10^{-28} cm^6/s$, therefore $I_{Auger} = q n_i^2 N_D \cdot d_a C = 6.6 pA/cm^2$.

We see that all non-radiative recombination currents are cumulatively smaller than the radiative recombination current $67 pA/cm^2$, as is typically the case for epitaxially-grown thin-film III-V photovoltaic cells, which therefore exhibit very high internal quantum efficiency[14,12,3,15].

Since the above analysis is quite approximate, in Figure S6a, we plot the efficiency of the system of Figure 2, when the recombination current is doubled from the value calculated using only radiative recombination, and we find that efficiency drops at most by 2.4%. Moreover, in practice the PV cell will likely include window layers for passivation, so the minority carriers will be decoupled from the electrodes and, no matter what their precise distribution inside the absorber may be, there should be little modification to their radiative recombination rates inside the ultra-thin absorber film compared to our simple assumption of constant quasi-Fermi levels. Therefore, we do not expect the efficiency error due our simplified electronic modeling and negligence of non-radiative recombination to exceed 3% in most cases discussed in this article.

An exception may be the case of 'opaque' electrodes: We conservatively limited the thickness of the 'convergence layers' to $0.005\lambda_g$, so that they are only a small perturbation to the real system. Then the optimal front 'convergence layer' is clamped at maximum thickness in Figure S1c in the 'opaque' frequency regime and in Figure S4g for the 'opaque' electrodes ITO and Ag. Instead, if we allow their thicknesses to be unlimited, in Figure S6, we show that a substantial increase in the 'opaque' efficiency is observed. This leads us to believe that the performance evaluation of 'opaque' electrodes may be more sensitive to precise electronic modeling of the pn junction.

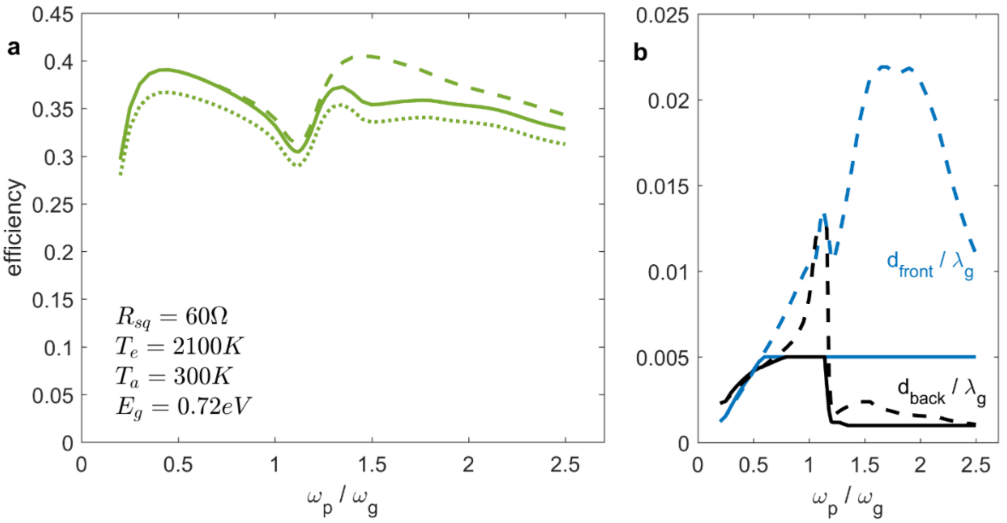

Supplementary Figure S6. Optimization results vs doping level ω_p for the structure of Fig.1 with model electrode $\hbar\gamma = 0.0072eV + 0.04\hbar\omega_p$, at $T_e = 2100°K$, $T_a = 300°K$, $R_{sq} = 60\Omega$ and with $E_g = 4k_B T_e = 0.72eV$, *with* upper limit $0.005\lambda_g$ for the 'convergence layers' (solid curves, same as those in Figs.2a and S1c) and *without* upper limit (dashed curves): **(a)** Efficiency η; allowing for thicker front 'convergence layer' results in significantly increased efficiency in the 'opaque' frequency regime ($\omega_p > \omega_g$), thus hinting that the 'opaque' results are quite sensitive to precise modeling of the electronic details of the pn-junction; also shown in dotted curve the efficiency when the recombination current is doubled compared to that in the solid curve, leading to a drop of at most 2.4%. **(b)** Normalized front and back 'convergence layer' thicknesses d_{front} and d_{back}.

REFERENCES

1. Dashiell, M. W. *et al.*, Quaternary InGaAsSb Thermophotovoltaic Diodes. *IEEE Transactions on Electron Devices* **53** (12), 2879 (2006).

2. Sze, S. M., *Physics of Semiconductor Devices*, 2nd ed. (Wiley, New York, 1981).

3. Steiner, M. A. *et al.*, Effects of internal luminescence and internal optics on Voc and Jsc of III-V solar cells. *IEEE Journal of Photovoltaics* **3** (4), 1437-1442 (2013).

4. Zeghbroeck, B. V. V., *Principles of Semiconductor Devices and Heterojunctions* (Prentice Hall, 2009).

5. DeSutter, J., Vaillon, R. & Francoeur, M., External Luminescence and Photon Recycling in Near-Field Thermophotovoltaics. *Physical Review Applied* **8** (1), 014030 (2017).

6. Lush, G. B. *et al.*, Microsecond lifetimes and low interface recombination velocities in moderately doped n-GaAs thin films. *Applied Physics Letters* **61** (20), 2440-2442 (1992).

7. Dawson, P. & Woodbridge, K., Effects of prelayers on minority-carrier lifetime in GaAs/ AlGaAs double heterostructures grown by molecular beam epitaxy. *Applied Physics Letters* **45** (11), 1227-1229 (1984).

8. Juntunen, M. A. *et al.*, Near-unity quantum efficiency of broadband black silicon photodiodes with an induced junction. *Nature Photonics* **10**, 777-782 (2016).

9. Repo, P. *et al.*, Effective Passivation of Black Silicon Surfaces by Atomic Layer Deposition. *IEEE Journal of Photovoltaics* **3** (1), 90-94 (2013).

10. Otto, M. *et al.*, Extremely low surface recombination velocities in black silicon passivated by atomic layer deposition. *Applied Physics Letters* **100** (19), 191603 (2012).

11. Olson, B. V. *et al.*, Time-resolved optical measurements of minority carrier recombination in a mid-wave infrared InAsSb alloy and InAs/InAsSb superlattice. *Applied Physics Letters* **101** (9), 092109 (2012).

12. Schnitzer, I., Yablonovitch, E., Caneau, C. & Gmitter, T. J., Ultrahigh spontaneous emission quantum efficiency, 99.7% internally and 72% externally, from AlGaAs/GaAs/ AlGaAs double heterostructures. *Applied Physics Letters* **62** (2), 131-133 (1993).

13. Walker, A. W. *et al.*, Impact of Photon Recycling on GaAs Solar Cell Designs. *IEEE Journal of Photovoltaics* **5** (6), 1636-1645 (2015).

14. Kayes, B. M. *et al.*, 27.6% conversion efficiency, a new record for single-junction solar cells under 1 sun illumination, *Proc. 37th IEEE Photovoltaic Spec. Conf., Seattle, WA, USA* (2011).

15. Tong, J. K., Hsu, W.-C., Huang, Y., Boriskina, S. V. & Chen, G., Thin-film 'Thermal Well' Emitters and Absorbers for High-Efficiency Thermophotovoltaics. *Scientific Reports* **5**, 10661 (2015).
